# Time-Dependent Effects of Rapid-Acting Antidepressants in iPSC-Derived Neurons from Treatment-Resistant Depression and Healthy Volunteers

**DOI:** 10.21203/rs.3.rs-8733841/v1

**Published:** 2026-02-12

**Authors:** Jenessa Johnston, Greg Jones, Shiyong Peng, Peixiong Yuan, Mani Yavi, Bashkim Kadriu, Ioline Henter, Brandi Quintanilla, Abdel G. Elkahloun, Ruin Moaddel, Anton Schulmann, Nirmala Akula, Mark Kvarta, Francis McMahon, Carlos Zarate

**Affiliations:** NIMH; National Institute of Mental Health; NIH; National Institute of Mental Health; NIMH-NIH; University of Texas Health Science Center at Houston; National Human Genome Research Institute; National Institute of Mental Health; National Institutes of Mental Health, NIH; National Institute of Mental Health/NIH; National Institute of Mental Health Intramural Research Program; National Institutes of Health; NIMH

## Abstract

Rapid-acting antidepressants like ketamine and serotonergic psychedelics show promise for treatment-resistant depression (TRD), but the molecular mechanisms that contribute to their therapeutic effects remain unclear. Induced pluripotent stem cells (iPSCs) offer a platform to model human cortical neurons and investigate drug effects in a human-relevant system. Here, iPSCs from individuals with TRD and healthy volunteers (HVs) were differentiated into mature cortical-like neurons and treated for six and 24 hours with agents being investigated as rapid-acting antidepressants, including (2*R*,6*R*)-hydroxynorketamine (HNK), psilocybin, lysergic acid diethylamide (LSD), and 2,5-Dimethoxy-4-iodoamphetamine (DOI). Bulk and single-cell RNA sequencing assessed global and cell-type-specific transcriptomic responses. Synaptic proteins were evaluated via Western blotting and immunocytochemistry. To validate translational relevance, transcriptomic results were compared to CSF proteomics from ketamine-treated HVs.

Despite differing initial pharmacological targets, overall gene expression across all compounds was highly correlated at matched timepoints compared to vehicle control, suggesting shared downstream effects. Both glutamatergic and serotonergic drugs converged on pathways involving inflammation, mTORC1 signaling, and cellular growth. At the single-cell level, HNK showed distinct cell-type specific alterations: upregulation in excitatory neurons and concomitant downregulation of inhibitory neuron populations. Differentially expressed genes from HNK-treated neurons also overlapped with CSF proteomic signatures from ketamine-treated individuals, supporting the model’s translational relevance. This study is the first to assess multiple putative rapid-acting antidepressants in parallel using an iPSC-derived neuron model. Both convergent and drug-specific changes in gene expression and pathway enrichment were observed across diverse compounds, supporting the use of human iPSC-derived neurons in antidepressant drug discovery.

**Clinical Trial Registry:**

www.clinicaltrials.gov, NCT02484456

## Introduction

Approximately one-third of individuals with major depressive disorder (MDD) have treatment-resistant depression (TRD), defined as lack of response to two or more conventional antidepressants such as selective serotonin reuptake inhibitors (SSRIs) [1]. Novel, rapid-acting antidepressants are urgently needed, but developing therapeutic targets using animal models has proven challenging. Determining molecular neural mechanisms within human-derived systems may more effectively propel drug discovery efforts [2].

The development of induced pluripotent stem cell (iPSC) technology has opened new avenues for studying neuropsychiatric conditions using patient-derived cells [3, 4]. iPSC modeling allows the examination of genetic contributions to disease mechanisms and cell type-specific therapeutic responses [5], capturing individual genetic variation that animal models cannot fully assess. Studies using iPSCs have yielded insights into treatment response mechanisms in depression, including altered serotonergic signaling in SSRI non-responders [6, 7].

The glutamatergic modulator ketamine has emerged as a promising rapid-acting antidepressant for TRD, improving symptoms within hours through increased α-amino-3-hydroxy-5-methyl-4-isoxazolepropionic acid receptor (AMPAR) transmission [8, 9]. (2*R*,6*R*)-hydroxynorketamine (HNK), a major ketamine metabolite essential to its antidepressant actions [9, 10], increases dendritogenesis and AMPAR transmission in rodent models [11]. iPSC studies of ketamine found increased neural progenitor proliferation via cAMP-IGF2 signaling [12] and enhanced structural plasticity via AMPAR-driven brain-derived neurotrophic factor (BDNF) and mechanistic target of rapamycin (mTOR) signaling [11], paralleling observations in animal models.

Serotonergic psychedelics (SPs) like psilocybin similarly demonstrate rapid and sustained antidepressant effects in TRD [13, 14]. Although they act on different primary receptors, psilocybin appears to converge with ketamine on common downstream mechanisms involving cortical glutamate release and synaptic plasticity via BDNF-mTOR signaling [15]. Other SPs, including lysergic acid diethylamide (LSD) and 2,5-Dimethoxy-4-iodoamphetamine (DOI), act through similar 5-HT2A receptor-dependent mechanisms [15, 16]. However, previous iPSC studies examined single agents only, and none incorporated cell lines from individuals with MDD or TRD.

Despite different primary receptor targets, ketamine and SPs appear to share clinical applicability across mood, anxiety, and substance use disorders, suggesting common transdiagnostic mechanisms [17]. This study used a consistent experimental framework in neurons derived from both TRD patients and healthy volunteers (HVs) to evaluate and compare time-dependent molecular changes induced by (2*R*,6*R*)-HNK, psilocybin, LSD, and DOI. To our knowledge, this is the largest sample of iPSC-derived neurons assessed with these putative rapid-acting antidepressants. As an exploratory validation, transcriptomic signatures from (2*R*,6*R*)-HNK were compared to CSF proteomic changes after ketamine infusion in a previously examined group of HVs.

## Materials and Methods

### Study participants

Peripheral blood mononuclear cells (PBMCs) were collected from five individuals with TRD enrolled in a crossover trial at the National Institutes of Health (NIH) Clinical Center (NCT02484456). Fibroblasts from five HVs were also collected for reprogramming following standard procurement procedures. All participants were female (age range = 20–68 years) in order to conserve statistical power within a small sample size. Further demographic information can be found in **Supplementary Table S1**. The study protocol was approved by the Institutional Review Board of the NIH, and written informed consent was obtained from all participants prior to any study procedures. The study was conducted in accordance with NIH ethical guidelines and the Declaration of Helsinki (2000).

### Generation of iPSC-derived neurons

iPSCs were reprogrammed at the National Heart, Lung, and Blood Institute (NHLBI/NIH) iPSC Core Facility using non-integrating approaches. No clonal abnormalities were detected, and a normal karyotype was confirmed across all cell lines. iPSCs were generated and differentiated into cortical neurons using established non-integrating reprogramming and neural induction protocols. Further details can be found in the Supplementary Methods. At 10 weeks, neurons differentiated under this protocol form synapses and have spontaneous synaptic activity alongside other neuronal markers of maturity [18]. All cell lines expressed pluripotency markers and were consistently assessed to be mycoplasma-free. At 10 weeks, the maturity of cortical neurons was confirmed via immunocytochemical staining for MAP2, post-synaptic density protein 95 (PSD-95), and β-tubulin III. A CCK-8 cell counting kit was used to determine viability.

### Drug treatment

At 10 weeks of maturity, primary cortical neurons were treated for six or 24 hours with acetonitrile vehicle (0.1%), (2*R*,6*R*)-HNK (1 uM), ketamine, LSD (10 uM), psilocybin (10 uM), or DOI (10 uM). All cell lines were exposed to each treatment and timepoint in replicate wells (n = 5/condition). A schematic of the experimental design appears in **Supplementary Figure S1**.

Justification of drug concentration and timepoints can be found in the Supplementary Methods. Expression of N-methyl-D-aspartate (NMDA) receptors and serotonin receptors was verified to determine whether the iPSC-derived forebrain neurons contained the canonical signalers for ketamine and psychedelic actions (**Supplementary Fig. S2**).

### Bulk RNA sequencing

RNA was isolated from treated cells, sequenced with the NovaSeq XPlus platform, and analyzed for differential gene expression using standard pipelines (STAR, featureCounts, Dream). Biological replicates (n = 2) were assessed for all treatments and timepoints across each cell line. Given the difference in cell lineage (fibroblasts and PBMCs), diagnosis was treated as a random effect in data processing to infer conclusions regarding treatment effects across cell lines. Significant contributors to variance as determined by VariancePartition [19], such as sequencing batch and subject, were also included as random effects. After removing lowly-expressed genes (genes with counts per million > 0.1 in at least five samples were included), differential gene expression analysis was conducted with Dream, a linear mixed model that is a variation of limma/voom analysis for assessing drug-treatment effects [20]. The cutoff for differentially expressed genes (DEGs) was set at an uncorrected p < 0.01 and logfold2 change > 1.5.

Batch corrections were applied as appropriate. De-identified data and analysis codes supporting the findings of this study are shared within the Open Science Framework (osf.io).

### Single-cell RNA sequencing

After 24-hour treatment, cells were processed for scRNA-seq using the 10x Genomics platform. scRNA-seq data were processed with Cellranger and Seurat (V5) in R [21, 22]. Initial quality control involved filtering cells based on median absolute deviations (MADs) to remove outliers [23]. For mitochondrial percentage, an upper threshold was set to filter cells with high mitochondrial reads (> 3 MAD). For genes and unique molecular identifiers (UMIs), cells were filtered that were either above or below three MADs. One sample (LSD from an HV) was excluded due to very high quantities of low complexity cells. For the remaining samples, the data were then integrated across samples using Canonical Correlation Analysis (CCA). Two clusters were subsequently excluded from downstream analysis. The first contained (7–49) cells across conditions, preventing statistically meaningful comparisons. The second exhibited clear signs of being driven by technical rather than biological variation (i.e., elevated mitochondrial and ribosomal percentages coupled with reduced gene and UMI counts). Because more stringent global filtering parameters resulted in excessive removal of cells from biologically relevant clusters, targeted cluster removal was used to maintain data integrity for downstream analysis. Manual annotation of integrated cell clusters was performed; details are provided in the Supplementary Methods.

### Western blotting

Protein lysates were analyzed by Western blot for synaptic and signaling proteins, with normalization to total protein (Ponceau); this method was used because typical housekeeping genes could potentially be expressed at different levels within TRD and HV cell lines. Prior to Western blotting, cells were homogenized in cold lysis buffer. Ten μg of protein were electrophoretically resolved in Mini-PROTEAN^®^ TGX Precast 4–12% gels (BioRad, Hercules, CA) then transferred onto nitrocellulose membranes via a semi-dry transfer method in the Trans-Blot Turbo Transfer System (BioRad, Hercules, CA). Further details are provided in the Supplementary Methods.

### Immunocytochemistry

Fixed neuronal cultures were stained for synaptic and neuronal markers, including synaptophysin, PSD-95, and MAP2. Statistical analyses were conducted in RStudio. A negative binomial regression model was conducted to examine the effects of treatment and timepoint on synapse count, controlling for diagnosis and subject as random effects. The dispersion parameter (3.11) suggested overdispersion, justifying the use of negative binomial regression over Poisson regression. Further details regarding imaging and processing can be found in the Supplementary Methods.

### Clinical comparison with CSF proteomics

DEGs from *in vitro* experiments were compared to CSF proteomics data from HVs post-ketamine infusion. HVs (mean age = 27 ± 6; F:M 4:5) were recruited in a separate study (NCT03065335) and had serial CSF collection after ketamine infusion. Details of the previous study can be found in [24].

Proteomics were performed using the Olink Explore platform (Inflammation I, Oncology I, Cardiometabolic I, and Neurology I reagent kits). Because the primary intention was to identify overlapping iPSC targets that correspond to the “ground-truth” (i.e., human CSF post-ketamine), and because of limited assay coverage of DEGs in the Olink platform (see Discussion), differential CSF protein expression was false discovery rate (FDR)-corrected for significance, while DEGs from the iPSC conditions were kept uncorrected (p < 0.05). To determine whether there was significant overlap between the patterns of differential CSF protein and iPSC gene expression over time, 22,810 genes tested for differential expression and the 1461 proteins that are able to be assessed by the Olink Explore platform (see Supplementary Table S5 in [24]) were used as background (n = 1235 overlapping analytes).

## Results

### Bulk RNA sequencing revealed overlapping and time-dependent effects of drug treatment

Assessing the neural molecular mechanisms of novel rapid-acting antidepressants such as (2*R*,6*R*)-HNK and SPs is a clear priority. To determine time-dependent effects, these therapeutics were applied to iPSC-derived cortical neurons from participants with TRD and HVs. Neurons were collected at six and 24 hours after treatment. Each agent was first compared to vehicle-control treated iPSC-derived neurons to determine time-dependent drug effects. In addition to differences from vehicle-control treated neurons, similarities and differences in drug treatment effects were categorized to ascertain overlapping and distinct mechanisms between drug types (glutamatergic and serotonergic). Expression of canonical ketamine/(2*R*,6*R*)-HNK and SP receptors was verified (**Supplementary Fig. S2**).

Differential expression analysis revealed significant alterations in gene expression across time and treatment groups (**Supplementary Fig. S3A**). At six hours, 718 DEGs were observed for (2*R*,6*R*)-HNK-treated neurons, 246 DEGs were observed for psilocybin, 299 DEGs were observed for LSD, and 493 DEGs were observed for DOI. A higher number of DEGs were observed at 24 hours ((2*R*,6*R*)-HNK = 790; psilocybin = 1419; LSD = 1822; DOI = 389). Because disparate and overlapping mechanisms between putative rapid-acting antidepressants are of great interest for future drug development, common DEGs between drug treatment groups were also assessed. As expected, the canonical SPs (psilocybin and LSD) had the most DEGs in common, with over 22.6% overlap at 24 hours (hypergeometric p < 2.2e-16, OR = 7.48).

A highly correlated directionality of log-fold changes between drugs at six and 24 hours was found when comparing genes that survived the p < 0.05 correction within any condition (n = 4325) (see **Supplementary Fig. S3B** for Spearman correlation values). Correlations across different drugs within the same timepoints were also high, particularly at 24 hours (r_s_ 0.74–0.83). Interestingly, correlations between the same drugs at the two different timepoints were negative, a reversal of transcriptomic effects that suggests response [25]. All DEG lists are presented in **Supplementary Tables S2-S9**.

**Figure 1:**
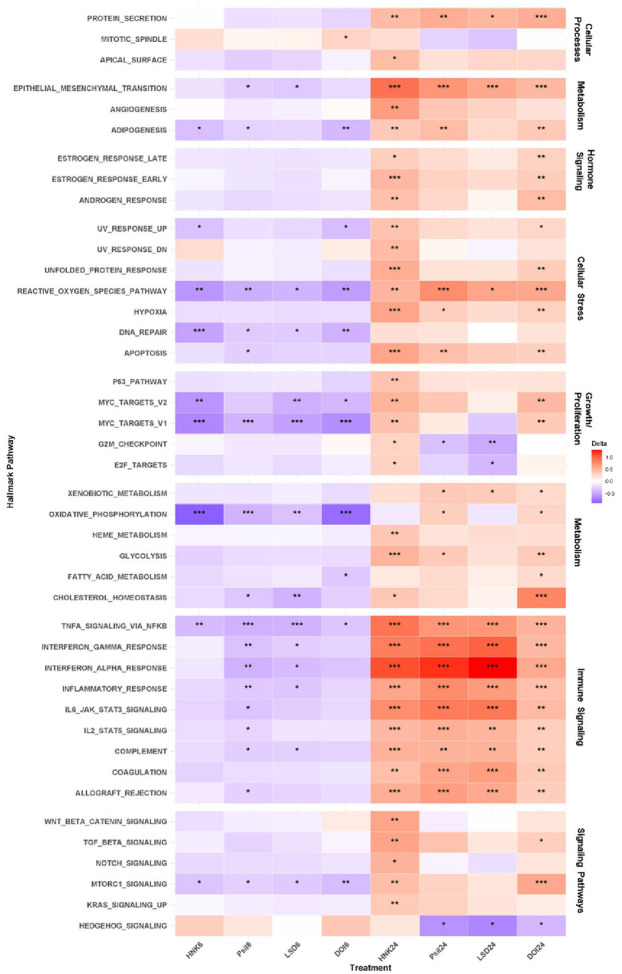
Most hallmark signaling pathways were downregulated at six hours and upregulated at 24 hours after all drug treatments. Data is from bulk RNA-sequencing analysis. Color scale indicates delta scores. Blue represents downregulated pathways and red represents up-regulated pathways compared to vehicle-treated controls. *p<0.05, **p<0.01, ***p<0.001 as false discovery rate (FDR)-corrected values.

Gene set enrichment analysis was conducted using the Molecular Signatures Database (MSigDB) as reference. Striking time-dependent effects were found in the activation of functional enrichment pathways. Downregulated signaling pathways at six hours were completely reversed at 24 hours, demonstrating primarily upregulated signaling. Across all drug treatments at six hours, many hallmark signaling pathways were downregulated compared to vehicle control, including oxidative phosphorylation (p < 0.01 in all treatment groups) and tumor necrosis factor alpha (TNF-α) signaling via nuclear-factor kappa beta (NFκβ) (p < 0.05 in all treatment groups). At six hours, changes in hallmark signaling pathways were most similar between psilocybin and LSD, and significant decreases in cholesterol homeostasis and immunomodulatory pathways were absent after treatment with DOI or (2*R*,6*R*)-HNK (Fig. 1).

However, at 24 hours, all drug effects were reversed; specifically, hallmark pathways involved in immune signaling, cellular metabolism, cellular stress, and canonical signaling were upregulated (Fig. 1). (2*R*,6*R*)-HNK had the most widespread impact across pathways, significantly upregulating 36 of 50 MSigDB hallmark signaling pathways at 24 hours. Interferon gamma and alpha response, interleukin-6 (IL6)-Jak-STAT3, IL2-STAT5, allograft rejection, and complement and coagulation pathways were upregulated at 24 hours across all treatments. mTOR complex 1 (mTORC1) signaling, often associated with rapid-acting antidepressant drug response, was decreased across all treatments at six hours but significantly upregulated at 24 hours by (2*R*,6*R*)-HNK and DOI (Fig. 1). Notably, hedgehog signaling was the only pathway significantly downregulated at 24 hours after all SP treatments. All FDR corrected p-values can be found in **Supplementary Table S10**.

Diagnosis was included as a random effect in all analyses given the difference in cell lineages between HV (fibroblast-derived) and TRD (PBMC-derived) samples; however, an exploratory analysis of diagnostic differences in hallmark signaling pathways was also conducted (see **Supplementary Fig. S4** and Supplementary Results). TRD cell lines had significantly lower expression of genes associated with growth and proliferation pathways, as well as signaling pathways such as Notch and Wnt/β-catenin.

### Single-cell RNA sequencing clustering and cell-type specific drug responses

Bulk RNA-seq provided insights into global transcriptional changes, but our iPSC-derived neurons comprised heterogeneous cell populations at various maturation stages. A comparative analysis of bulk and scRNA-sequencing data can be found in the Supplementary Results. Drug effects are known to be highly cell type- and maturation-dependent, especially for ketamine and 5-HT2A-targeting agents [26, 27]. Because changes in excitatory/inhibitory (E/I) balance also correlate with rapid antidepressant effects [28, 29], scRNA-seq with annotation focused on cell type (excitatory vs. inhibitory) and maturation stage (pseudotime) was used to identify selective drug targets.

**Figure 2:**
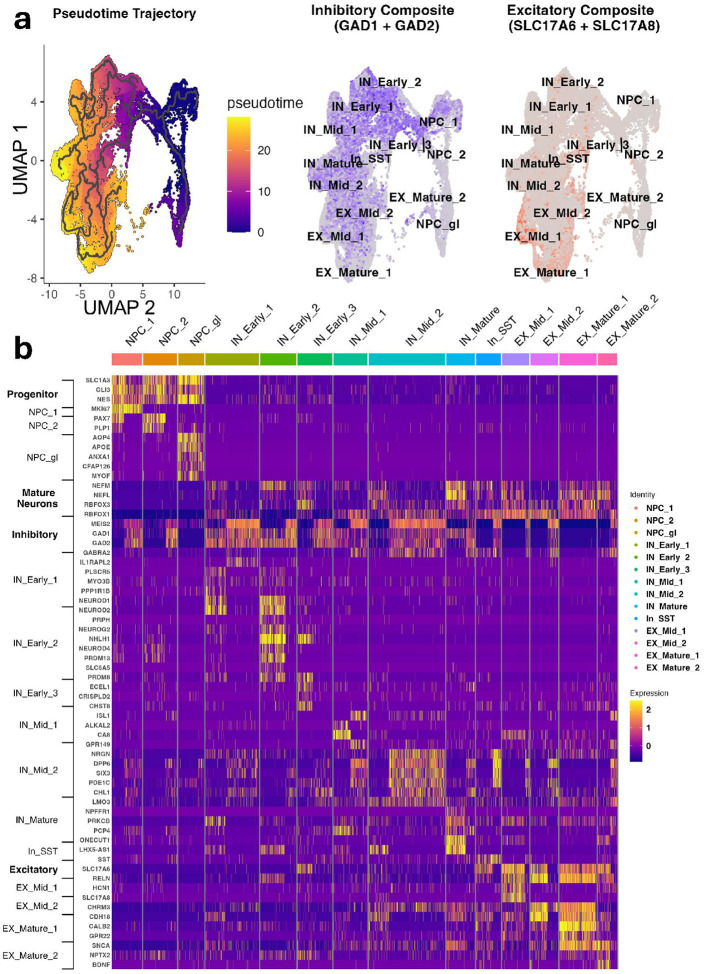
Single cell analysis of iPSC-derived neurons revealed distinct excitatory and inhibitory neuronal populations. **(A)** Uniform Manifold Approximation and Projection (UMAP) embeddings of single cell clusters showing differentiation stages across clusters (pseudotime) and distribution of inhibitory (left panel, GAD1+GAD2 composite, blue) and excitatory (right panel, SLC17A6+SLC17A8 composite, red) neuronal populations. The plot shows distinct separation of neural progenitor cells (NPCs), inhibitory neurons (INs), and excitatory neurons (EXs) at various maturation stages. Composite scores for canonical inhibitory/excitatory genes were generated using the AddModuleScore function in Seurat. Pseudotime was performed with Monocle3. **(B)** A heatmap of cell type-specific marker genes across identified clusters showed differential expression patterns of canonical neuronal markers and cluster-specific genes identified via cosine similarity from COSG in R. Ranked median pseudotime values per cluster were used to subdivide clusters into early, mid, and mature types within the excitatory and inhibitory populations. Notable markers included MKI67 for proliferative neural progenitors (NPC_1), AQP4/APOE for glial-fated progenitors (NPC_gl), GAD1/GAD2 for inhibitory neurons, and SLC17A6/SLC17A8 for excitatory neurons. The color gradient represents normalized expression levels from 0 (low, purple) to 2 (high, yellow).

iPSC-derived neurons were treated with vehicle, (2*R*,6*R*)-HNK, psilocybin, or LSD for 24 hours before single-cell sequencing. After quality control and data integration, unsupervised clustering identified 13 cell populations, annotated using canonical marker genes and developmental trajectory analysis (**Supplementary Table S11**). These clusters were grouped into neural progenitor cells (NPCs), inhibitory neurons (INs), and excitatory neurons (EXs), each further subdivided by maturation stage. Drug treatments were proportionally distributed across clusters, enabling assessment of drug effects by cell type and maturation (**Supplementary Fig. S5**). Distinct populations of excitatory and inhibitory neurons were identified based on canonical marker expression. Inhibitory clusters showed strong *GAD1* and *GAD2* expression, while excitatory clusters expressed glutamate transporters *SLC17A6* and *SLC17A8* (Fig. 2A).

Additional annotation details, including conformation of cortical neuronal phenotype (**Supplementary Figs. S6, S7**) appear in the Supplementary Results.

### Serotonergic psychedelics showed broad pathway modulation across mature neuronal populations

**Figure 3:**
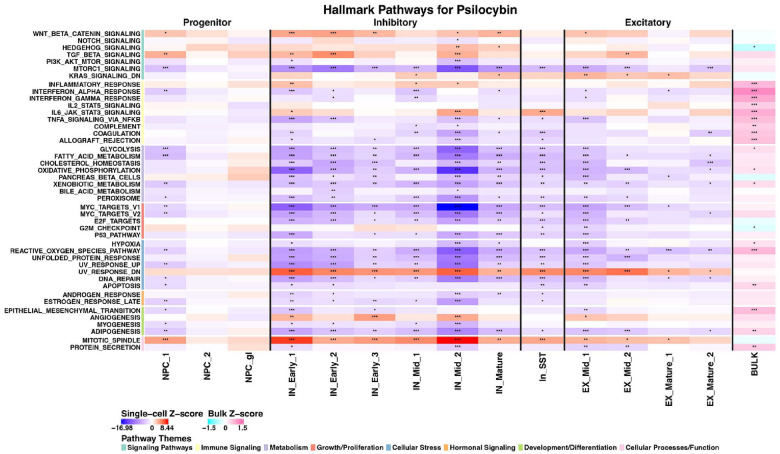
Cell-type specific pathway modulation by psilocybin at 24 hours. Heatmap showing hallmark pathway enrichment across neural cell clusters after psilocybin treatment. Color intensity indicates combined z-score enrichment from model-based analysis of single-cell transcriptomics (MAST) bootstrapped gene set enrichment analysis (GSEA) (red = upregulation, blue = downregulation). For comparison, the final column (right) contains the bulk differential expression z-scores from zenith (pink = upregulation, cyan = downregulation). Psilocybin showed broader effects across multiple mature neuronal populations, with pronounced activity in inhibitory neurons. Note the significant downregulation of pancreas beta cells and E2F target pathways across multiple inhibitory clusters and strong upregulation of transforming growth factor-beta (TGF-beta) signaling, angiogenesis, and IL6-JAK-STAT3 signaling in IN_Early_1, IN_Mid_1, and IN_SST clusters. “UV response down” was consistently upregulated across most neuronal clusters. Asterisks indicate significance level: * p<0.05, ** p<0.01, *** p<0.001.

**Figure 4:**
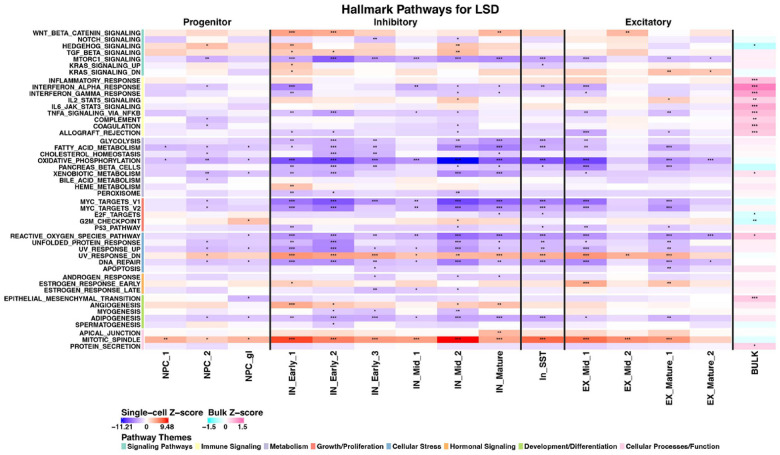
Cell-type specific pathway modulation by LSD at 24 hours. Heatmap showing hallmark pathway enrichment across neural cell clusters after treatment with LSD. Color intensity indicates combined z-score enrichment from model-based analysis of single-cell transcriptomics (MAST) bootstrapped gene set enrichment analysis (GSEA) (red = upregulation, blue = downregulation). For comparison, the final column (right) contains the bulk differential expression z-scores from zenith (pink = upregulation, cyan = downregulation). Notable features include significant downregulation of oxidative phosphorylation, MYC targets, and hedgehog signaling across multiple inhibitory clusters, with upregulation of IL2-STAT5 signaling and “UV response down”. “Estrogen response late” was upregulated in IN_SST and several other mature neuronal clusters. Asterisks indicate significance level: *p<0.05, **p<0.01, ***p<0.001.

Examination of hallmark pathway enrichment across cell clusters revealed that psilocybin and LSD exhibited remarkably similar pathway signatures (Figs. 3 & 4). Both SPs demonstrated significant pathway alterations predominantly in mature neuronal populations, with a slight bias towards inhibitory neurons. The most pronounced effects were observed in the IN_Mid_1, IN_Mid_2, and IN_Mature clusters. In the inhibitory populations, both psilocybin and LSD strongly upregulated pathways associated with cellular proliferation, including mitotic spindle (particularly pronounced in IN_Mid_2) and KRAS signaling. Concurrently, both drugs activated neuroprotective mechanisms by upregulating transforming growth factor beta (TGF-β) signaling across multiple inhibitory clusters. This pattern of enhanced proliferative and neuroprotective signaling may represent a core mechanism underlying the neuroplasticity-enhancing effects of SPs. Notably, both SPs significantly downregulated several metabolic pathways across inhibitory neurons, including glycolysis, fatty acid metabolism, and oxidative phosphorylation. This metabolic suppression was particularly evident in IN_Early_1, IN_Early_2, and IN_Early_3 clusters. The “pancreas beta cells” pathway was also consistently downregulated across multiple inhibitory clusters, suggesting altered energy metabolism regulation.

Inflammatory signaling pathways showed a complex pattern of regulation. Both psilocybin and LSD significantly downregulated TNFα signaling via NFκB, interferon alpha response, and interferon gamma response in inhibitory neurons (particularly IN_Early_1 and IN_Early_2). However, IL2-STAT5 signaling was upregulated for LSD (IN_Mid_2 and EX_Mature_1), and IL6-JAK-STAT3 signaling (IN_Mid_1, IN_SST) and inflammatory response (IN_Early_1, IN_Mid_1, IN_Mid_2) were upregulated for psilocybin, suggesting differential regulation of specific inflammatory pathways.

Cellular stress response pathways, including reactive oxygen species, unfolded protein response, and UV response, were predominantly downregulated across inhibitory clusters, suggesting that SPs may enhance neuronal resilience by reducing stress-related signaling. This effect was particularly pronounced in the IN_Early_1 and IN_Early_2 clusters. In excitatory neurons, both SPs showed less pronounced effects compared to inhibitory populations, though significant pathway alterations were observed in the EX_Mid_1 and EX_Mature_1 clusters. Notably, angiogenesis and epithelial-mesenchymal transition pathways were upregulated in EX_Mature_1 and EX_Mature_2, potentially reflecting enhanced structural plasticity mechanisms.

### (2R,6R)-HNK demonstrated highly specific cell-type targeting with bidirectional pathway regulation

**Figure 5:**
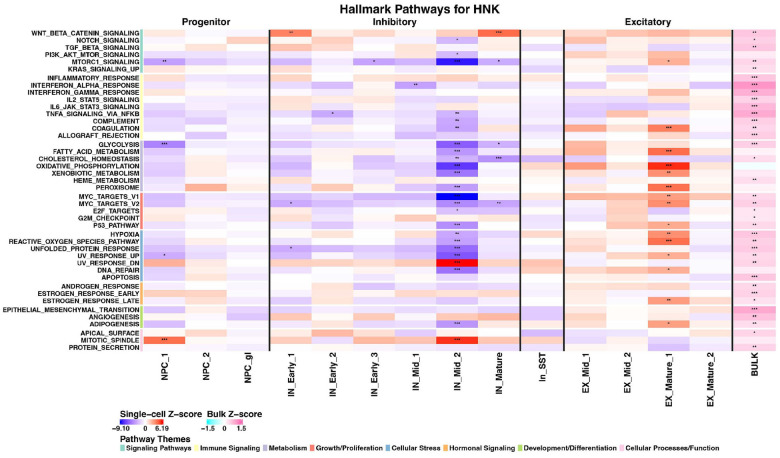
Cell-type specific pathway modulation by (2*R*,6*R*)-HNK at 24 hours. Heatmap showing hallmark pathway enrichment across neural cell clusters after (2*R*,6*R*)-HNK treatment. Color intensity indicates combined z-score enrichment from model-based analysis of single-cell transcriptomics (MAST) bootstrapped gene set enrichment analysis (GSEA) (red = upregulation, blue = downregulation). For comparison, the final column (right) contains the bulk differential expression z-scores from zenith (pink = upregulation, cyan = downregulation). (2*R*,6*R*)-HNK had highly specific effects primarily in EX_Mature_1 and IN_Mid_2 clusters, with bidirectional regulation of metabolic and signaling pathways. Note the striking upregulation of oxidative phosphorylation, fatty acid metabolism, MYC targets, and mTORC1 signaling in EX_Mature_1, with concurrent downregulation of these same pathways in IN_Mid_2. Asterisks indicate significance level: *p<0.05, **p<0.01, ***p<0.001.

In contrast to the broad effects of SPs, (2*R*,6*R*)-HNK exhibited high cell-type specificity, predominantly affecting only two neuronal populations: one inhibitory (IN_Mid_2) and one excitatory (EX_Mature_1) (Fig. 5). This targeted pattern of activity suggests a precise mechanism of action that may underlie its distinct therapeutic profile. (2*R*,6*R*)-HNK induced bidirectional regulation of several key pathways between these two clusters. Pathways governing metabolism, growth, and proliferation, including mTORC1 signaling, oxidative phosphorylation, fatty acid metabolism, glycolysis, and MYC target pathways (V1 and V2) were significantly upregulated in the excitatory EX_Mature_1 cluster and simultaneously downregulated in the inhibitory IN_Mid_2 cluster. Cellular stress pathways, including reactive oxygen species, UV response, and unfolded protein response were also regulated in opposite directions between these two clusters. The excitatory EX_Mature_1 cluster showed upregulation of these pathways, potentially reflecting increased metabolic activity, while the inhibitory IN_Mid_2 cluster showed downregulation, suggesting reduced cellular stress and metabolism. This mutual shifting mirrors the known mechanistic correlates of ketamine and its metabolites in humans [29] and may act to restore cellular balance to neuronal E/I.

Further KEGG pathway analysis of these (2*R*,6*R*)-HNK-responsive clusters revealed significant enrichment for “Morphine Addiction” and “Neuroactive Receptor-Ligand Interaction” pathways in both clusters, with the inhibitory cluster additionally showing enrichment for “Oxytocin Signaling”. These pathways appeared relatively specific to the (2*R*,6*R*)-HNK responsive clusters, with only two other cell types (IN_Early1, In_SST) showing similar pathway enrichment patterns (**Supplementary Figs. S8, S9**).

Together, these findings suggest that while both SPs and glutamatergic modulators ultimately converge on increased synaptic plasticity, they appear to engage distinct cellular targets and molecular pathways to achieve these effects. The cell-type specificity of (2*R*,6*R*)-HNK suggests a highly targeted mechanism of action, while the broader activity of SPs across multiple cell types may explain their unique subjective and therapeutic profiles.

### Protein expression changes revealed similar time-dependent changes after drug exposure

Synaptic-specific and immediate early genes, such as PSD-95 and mTORC1, which have previously been implicated in putative rapid-acting antidepressant response [9], were measured through Western blotting to determine if time-dependent changes paralleled the bulk RNA-sequencing as well as previous research in animal models. In addition, the treatment- and time-dependent effects found on mTORC1 signaling within the bulk and single-cell data warranted further exploration of related protein signalers. All significant results are presented in **Supplementary Table S12** with Tukey Honestly Significant Difference (HSD) values for post-hoc analyses compared to vehicle control. Other significant comparisons between drug treatments, rather than comparison to vehicle, are noted in the text.

Total ERK, an indicator of cell survival, was affected by treatment and by the interaction of time and treatment, with (2*R*,6*R*)-HNK significantly increasing total ERK expression from vehicle and DOI at 24 hours (**Supplementary Fig. S10A**). Phospho-eukaryotic translation elongation factor 2 (eEF2) was significantly impacted by treatment and interaction effects. Tukey’s post-hoc tests revealed significant differences between (2*R*,6*R*)-HNK and LSD treatment at six hours (p = 0.031) as well as significant increases in phospho-eEF2 expression compared to vehicle after (2*R*,6*R*)-HNK treatment at 24 hours (**Supplementary Fig. S10B**). Similarly, total eEF2 expression was increased by (2*R*,6*R*)-HNK at 24 hours (**Supplementary Fig. S10C**). Total eIF4E expression was increased by treatment, whereas phospho-eIF4E expression had significant interaction effects. Post-hoc testing revealed that (2*R*,6*R*)-HNK increased levels from vehicle control at 24 hours for both phospho-eIF4E and total eIF4E (**Supplementary Figs. S10D & S10E**).

Total 4EBP1 was upregulated by LSD compared to (2*R*,6*R*)-HNK at six hours (p = 0.016) (**Supplementary Fig. S10F**). PSD-95 was similarly affected, with LSD increasing PSD-95 expression from (2*R*,6*R*)-HNK at six hours (p = 0.047) and (2*R*,6*R*)-HNK increasing PSD-95 levels from both vehicle control and DOI (p < 0.001) at 24 hours (**Supplementary Fig. S10G**). Synaptotagmin was increased at 24 hours by (2*R*,6*R*)-HNK compared to DOI (p = 0.001) (**Supplementary Fig. S10H**). While a significant interaction between time and treatment was noted for Synapsin I, post-hoc testing revealed no specific differences. No significant differences were observed in levels of Dab1, phospho-ERK, GluA1, phospho-mTOR, mTOR, NR2B, phospho-4EBP1, phospho-TrkB, or TrkB.

### Synaptic counts were increased after drug treatment at 24 hours

Given the increase in synaptic-specific proteins and transcriptomic changes in mTORC1 signaling and protein secretion, immunocytochemistry was conducted to ascertain histological changes after drug treatment. Immunofluorescent co-expression of PSD-95, MAP2, and synaptophysin was used as a proxy measure to assess synapse count on neurons. The negative binomial regression model demonstrated good fit and all fixed effects were statistically significant (p<.001). The model also revealed significant treatment × timepoint interactions (p<.001), indicating that treatment effects varied across timepoints. At six hours, LSD and psilocybin significantly decreased synapse count compared to vehicle (p<.001). At 24 hours, all treatments significantly increased synapse count compared to vehicle (p < 0.001), with psilocybin showing the largest effect (b = 1.63, p<.001) and LSD the smallest (b = 0.34, p<.001) (**Supplementary Fig. S11**).

### Changes in iPSC-derived neurons mirrored those of CSF proteomics after ketamine treatment

The relative novelty of iPSC-based modeling for depression raises a pertinent question of comparability to living human neural processes. Preliminary evidence from our laboratory and others previously suggested that (2*R*,6*R*)-HNK’s NMDA-independent effects may drive ketamine’s longer-term antidepressant activity [10, 30]. Here, iPSC-based genetic responses to proteomic data were assessed from CSF collected over 24 hours (six timepoints) after a racemic ketamine infusion (0.5mg/kg over 40 minutes) in a previous study of nine HVs [24]. The present exploratory analysis sought to identify overlapping DEGs (uncorrected p < 0.05) between the (2*R*,6*R*)-HNK-treated iPSC neurons and the serial CSF proteomic data.

**Figure 6:**
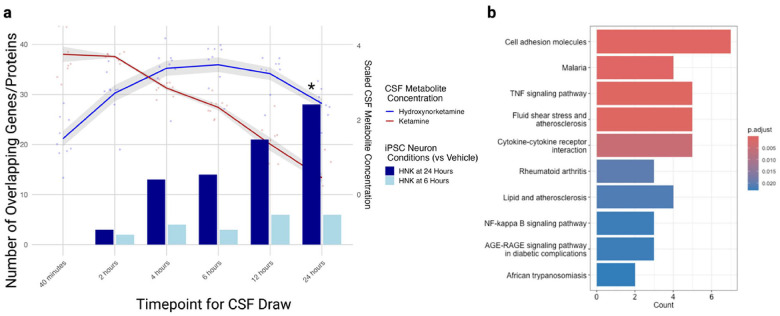
Overlap of significant differentially expressed genes (DEGs) and proteins. **(A)** Number of significantly overlapping genes from the six- and 24-hour timepoints. (2*R*,6*R*)-HNK iPSC-derived neuronal conditions compared to differentially expressed protein (from healthy volunteers (HVs) receiving IV racemic ketamine); proteomics were performed across serial CSF draws (six timepoints over 24 hours). Superimposed lines represent scaled CSF metabolite concentrations for ketamine (red) and (2*R*,6*R*)-HNK (blue) for the nine participants at each timepoint. HNK at 24 hours showed significant, overlapping enrichment with ketamine CSF expression at 24 hours (*10,000 permutation test; p=0.043). **(B)** KEGG-pathway enrichment for overlapping genes/proteins (n=28).

As shown in Fig. 6, differential gene expression after six-hour (2*R*,6*R*)-HNK exposure in neurons demonstrated minimal overlap or discernible patterns with CSF protein expression. In contrast, gene expression associated with (2*R*,6*R*)-HNK at 24 hours aligned progressively with CSF protein profiles, peaking at the equivalent 24-hour timepoint, which coincided with the observed maximal antidepressant effects that follow a single ketamine infusion [8]. Given this trend, a hypergeometric test was conducted to assess significant enrichment between the DEGs from (2*R*,6*R*)-HNK-treated iPSC neurons (24-hour condition) and differentially expressed proteins from the matched 24-hour CSF draw. Seventy-two iPSC-derived neuronal genes and 364 CSF proteins were differentially expressed, and 28 of them overlapped, indicating a nominally significant enrichment (permutation test: p = 0.043). Twenty-five of 28 were differentially expressed in the same direction in both datasets, far above what would be expected by chance after factoring in the proportion of up- and down-regulated genes in the background for both datasets (P_(X > 0.52)_ = 1.3e–5). Spearman’s rank correlations revealed a nonsignificant relationship for the 28 overlapping genes/proteins at the 24-hour CSF draw (ρ = 0.34, p = 0.07; FDR = 0.099), which was stronger for the 21 overlapping genes/proteins at 12 hours (ρ = 0.61, p = 0.004; FDR = 0.02). As can be seen in Fig. 6, these observations comported with relative concentrations of both ketamine (red line) and (2*R*,6*R*)-HNK (blue line) observed in the CSF of all nine IV ketamine recipients in the previous study [24]— correlations were strongest at the peak (2*R*,6*R*)-HNK concentration interval (Fig. 4). Consistent with overall trends in the bulk RNAseq pathway analysis, inflammatory cytokine signaling was significantly enriched in the KEGG pathway analysis.

The same analysis was repeated for each cell type cluster of the single cell data. DEGs were assessed after correction (p_fdr_<0.05). No individual cell types showed significant enrichment after correction (all p > 0.05). Ranked correlation for expression levels revealed a modest inverse relationship (ρ=−0.25; p_fdr_=0.073) for the EX_Mature_1 cluster (n = 124 genes/proteins). KEGG enrichment for this cluster returned one term: “Cell Adhesion Molecules” (n = 15 genes; p_fdr_=0.015). Four differentially expressed genes/proteins were common to all three datasets: CD276 (*B7-H3*), CD112 (*NECTIN2*), P4HB (*PDIA1*), and GRN (progranulin). Most DEGs (> 90%) overlapping with CSF were upregulated for our single cell data across clusters. STRING analysis was then performed for protein-protein interactions on the group of upregulated genes across all single-cell clusters that showed directional agreement with differentially expressed CSF proteins at the 24-hour timepoint (n = 102 proteins/genes). Using STRING’s in-built gene ontology enrichment, the top three molecular processes identified were insulin-like growth factor receptor activity (GO:0005010), insulin-like growth factor binding (GO:0005520), and neurotrophin receptor activity (GO:0005030) (all FDR < 0.05) (**Supplementary Fig. S12**).

## Discussion

This study is the first to report the effects of (2*R*,6*R*)-HNK and SPs in an iPSC-based neuronal model system. The significant positive correlations of gene expression changes across all drugs—but not between time conditions—suggests overlapping downstream mechanisms previously hypothesized to promote rapid antidepressant effects [31].

Bulk RNA-sequencing revealed large time-dependent effects across all treatments, with hallmark signaling pathways generally downregulated at six hours and up-regulated at 24 hours. Immune signaling (TNF-α via NF-κβ, interferon gamma/alpha response, IL6-Jak-STAT3, complement pathways) was upregulated across treatments at 24 hours. Interestingly, while peripheral inflammation is elevated in depression [32], the effects of ketamine and SPs on these systems appeared mixed [33]. Another study found increased proinflammatory signals in the CSF 24 hours post-ketamine infusion in HVs [24], paralleling our iPSC findings. Classic inflammatory signaling pathways such as IL-6-STAT3 may flip to neuroprotective phenotypes in the CNS, in contrast with their effects in the periphery [34, 35].

mTORC1 signaling was significantly downregulated at six hours across all treatments but upregulated at 24 hours by (2*R*,6*R*)-HNK and DOI, coinciding with increased synapse number and cellular outgrowth markers. This complex temporal pattern warrants further exploration in human iPSC models—particularly given paradoxical findings that the mTOR inhibitor rapamycin prolonged ketamine’s antidepressant effects in a two-week follow-up [36]. Extending ketamine’s clinical effects by administering rapamycin suggests that dampening mTORC1 signaling may help modulate neuroinflammation and stabilize synapses newly formed after the administration of rapid-acting antidepressants. The upregulation of mTORC1 at 24 hours, the increases in synapse number and other indicators of cellular outgrowth, and the increased inflammation observed here further align with mTORC1’s known promotion of inflammation. The complex role of mTORC1 warrants further exploration in *in vitro* models such as this, which allow for neuronal cell resolution in participants. In addition, the preliminary exploration of diagnostic differences revealed similar time-dependent effects as well as diagnosis-dependent effects on inflammatory and proliferative signaling that should be assessed further without confounding cell lineages.

At the single-cell level, drug effects predominantly occurred in mature neuronal clusters, suggesting that differentiation time is critical for future iPSC studies of these compounds. Psilocybin and LSD showed substantial transcriptional overlap, consistent with their mechanistic similarities [37]. Both agents upregulated inflammatory signaling (IL6-Jak-STAT3 (LSD), IL2-STAT5 (psilocybin), and TGF-β (both)), suggesting compensatory pro- and anti-inflammatory responses.

Notably, TGF-β signaling may serve as an instructive signal for neurons to switch from a “growth state” to a “synaptogenic state” [38, 39]—in line with downregulation of insulin-responsive genes (i.e., “hallmark pancreas beta cells”) with both SPs. In contrast to SPs, insulin signaling upregulation is a hallmark of treatment with ketamine and (2*R*,6*R*)-HNK [40–42]. For instance, STRING analysis showed enrichment for insulin-like growth factor receptor activity with (2*R*,6*R*)-HNK, and CSF insulin levels rose three- to four-fold at 12–24 hours post-ketamine [24]. This divergence suggests that while ketamine and SPs may achieve similarly rapid antidepressant effects, they may have distinct metabolic/energetic profiles. Whether this is related to why SPs impart a more durable clinical response than ketamine—even when psychotherapy is minimized [43, 44]—remains a testable and potentially impactful hypothesis going forward.

(2*R*,6*R*)-HNK activity was restricted to two cell clusters, with metabolic pathways (mTOR, OXPHOS, β-oxidation) downregulated in IN_Mid_2 and upregulated in EX_Mature_1. These clusters were enriched for neuroactive ligand-receptor interactions, opioid, and oxytocin signaling—all established ketamine mechanisms [33, 45, 46]. The pattern of expression also fits the E/I balance hypothesis well [47]. While such findings should not be over-interpreted in the context of our limited sample size, this pattern fits with both ketamine’s canonical plasticity-inducing and metabolic signals [33, 48], as well as with the E/I balance hypothesis underlying its most robust clinical biomarker, namely increased magnetoencephalographic γ-power [47].

Finally, Western blotting confirmed time-dependent changes in synaptic proteins (PSD-95) and increased phospho-eEF2, total eEF2, eIF4E, and phospho-eIF4E by (2*R*,6*R*)-HNK, implicating protein synthesis upregulation and translation mechanisms that parallel ketamine’s [49, 50]. Immunocytochemistry confirmed increased synapse counts after all treatments at 24 hours, supporting the hypothesis that cortical synapse increases are crucial for rapid-acting antidepressant effects [51].

Despite these intriguing findings, several limitations warrant attention. First, different starting lineages prevented diagnostic comparisons, though iPSC lines from the same donor were highly similar regardless of source cell type [52]. In addition, the nature of reprogramming somatic cells to iPSCs strips away epigenetic effects that may deeply impact the molecular pathology of TRD and the effects of various treatments. Future research should attempt to identify and mirror common epigenetic changes within TRD. Another limitation is the use of psilocybin rather than psilocin, though psilocybin is rapidly converted via alkaline phosphatase (*ALPL*) [53], which is an iPSC marker gene and was thus highly expressed in our sample (68th percentile of all expressed genes by mean CPM). While we had no direct evidence of *in vitro* psilocybin metabolism in our cell lines, the dephosphorylation of psilocybin has been observed within the human brain and across species, even those that do not express canonical enzymes such as *ALPL* [54]. Bulk RNA-sequencing and scRNA-sequencing diverged, and low sample size within the single-cell dataset may have contributed to observed differences. However, scRNA-sequencing analysis also showed strong cell-type specific changes, and it is possible that certain cell populations drove most of the overall transcriptomic changes. It should also be noted that the significant overlap between iPSC gene expression and CSF protein expression after (2*R*,6*R*)-HNK and ketamine treatment, despite different experimental modalities and donors, suggests that iPSC models may accelerate understanding of acute drug activity.

In sum, this study is the first comprehensive comparison of ketamine, (2*R*,6*R*)-HNK, and SPs in iPSC-derived neurons from TRD participants and HVs. Cell-type specific and time-dependent changes revealed convergent downstream mechanisms despite different receptor targets, with increased synaptic protein expression and outgrowth at 24 hours. Synaptic-specific protein expression and synaptic outgrowth were found at 24 hours post-treatment. The significant time-dependent overlap found between CSF protein expression and gene expression in our iPSC-derived neurons after treatment suggests that this model may provide direct insight to drug effects in patients, providing a promising platform for developing future rapid-acting antidepressants.

## Supplementary Files

This is a list of supplementary files associated with this preprint. Click to download.

- JohnstonetaliPSCDerivedNeuronsSUPPLTABLESFINAL.docx
- iPSCderivedneuronsHNKandSPsforMolPsychSUPPLFINAL12826.docx
